# From confusion to conclusion: the value of confocal microscopy in diagnosing chondroid syringoma^☆^

**DOI:** 10.1016/j.abd.2026.501312

**Published:** 2026-03-26

**Authors:** Nelson Lobos-Guede, Fernanda Jesús Ramírez-Bravo, Sofía Jiménez-Castillo, Ingrid Plass, Magdalena Delgado, Alex Castro

**Affiliations:** a Department of Dermatology, Faculty of Medicine, Clinica Alemana, Universidad del Desarrollo, Santiago, Chile; b Department of Cutaneous Oncology, Instituto Nacional del Cancer, Santiago, Chile; c Department of Head and Neck Surgery, Faculty of Medicine, Clínica Alemana, Universidad del Desarrollo, Santiago, Chile; d Department of Pathology, Faculty of Medicine, Clinica Alemana, Universidad del Desarrollo, Santiago, Chile

Dear Editor,

Cutaneous Mixed Tumor (CMT), or chondroid syringoma, is a rare and generally benign cutaneous adnexal tumor, accounting for 0.01% of all primary skin neoplasms.^1^ It may arise from both apocrine and eccrine glands.^2^ It is more frequently found in middle-aged men, commonly affecting the head and neck.^1^, ^3^ Clinically, CMT presents as a slow-growing, painless intradermal or subcutaneous nodule without ulceration, often evolving over several months (average 2‒12 months).^1^, ^2^ While its clinical and histopathological features are well documented, dermoscopic patterns have been scarcely described, and there are no previous reports using in vivo Reflectance Confocal Microscopy (ivRCM) to date.

We present the case of a 42-year-old woman who presented with a six-month history of a progressively enlarging, asymptomatic nodule on her nose. On physical examination (Fig. 1), an exophytic pinkish erythematous nodule with a sessile base was found on the right nasal sidewall. Dermoscopy (Fig. 2) revealed a polychromatic pattern with whitish, pink, and light brown homogeneous areas, associated with linear telangiectasias.

**Fig. 1:**
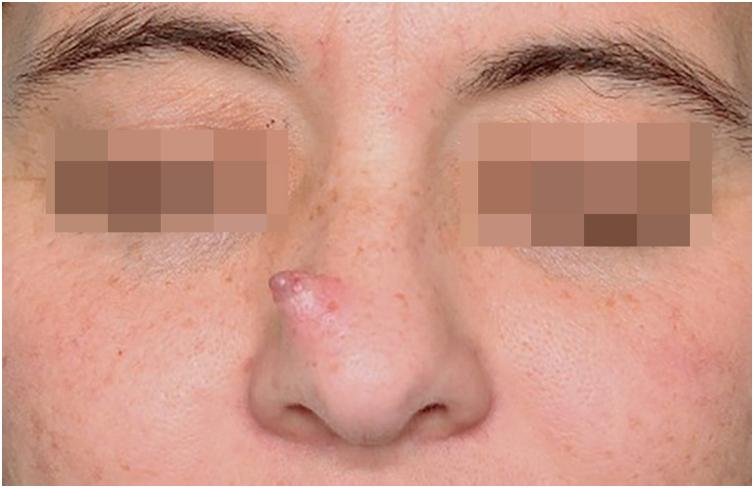
Clinical image of exophytic tumor with sessile, pinkish erythematous, well-demarcated base on the right lateral nasal slope.

**Fig. 2:**
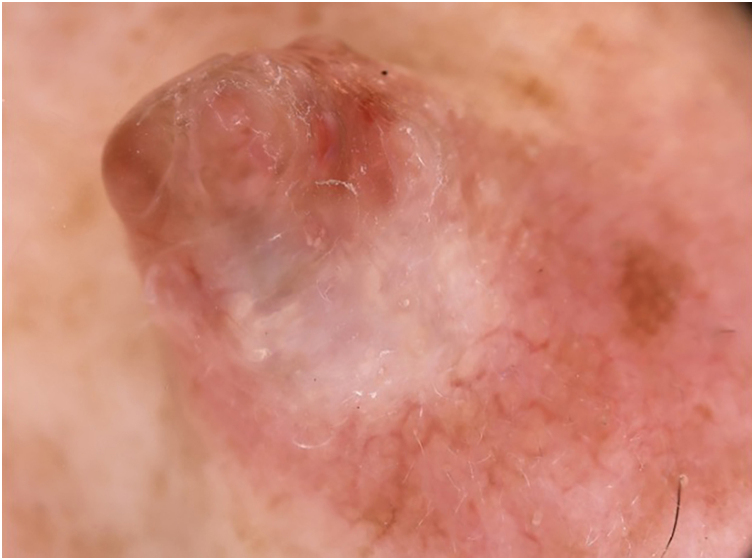
Dermoscopic image with 20× magnification. Polychromatic pattern with whitish, pink, and light brown homogeneous areas associated with linear telangiectasias.

The ivRCM (VivaScope® 3000) examination revealed a symmetrical honeycomb pattern in the epidermis, and a proliferation of homogeneous “pea-shaped” round brilliant cells forming oval structures (Fig. 3A‒D) was observed. Excisional biopsy showed a dermal proliferation formed by abundant chondroid and myxoid stroma in which epithelial cells are arranged in cords, nests, and isolated cells with ovoid nuclei, thin chromatin, small nucleoli, and pale eosinophilic cytoplasm. Foci of infundibular keratinization were also observed.

**Fig. 3:**
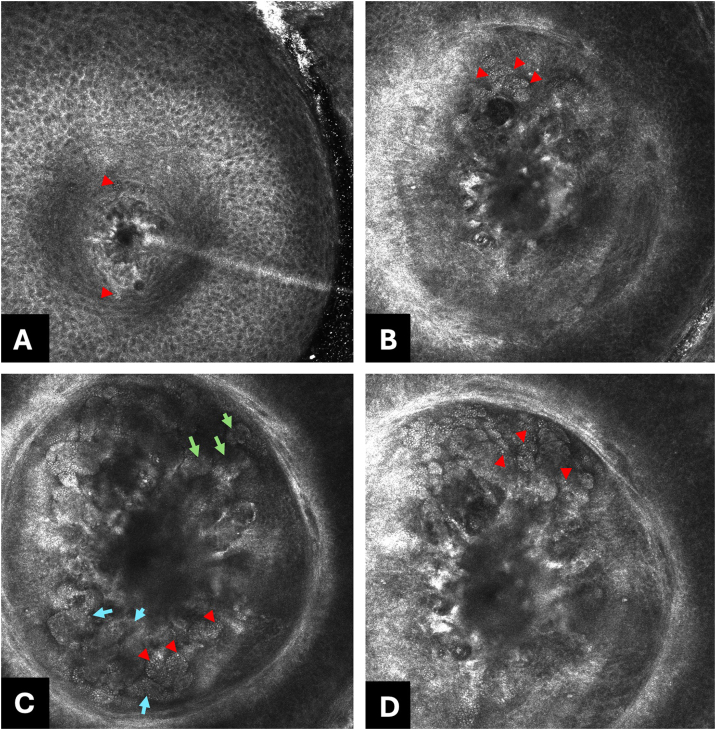
Reflectance in vivo Confocal Microscopy (RCM) individual images (500 × 500 μm) reveal: (A) Epidermal assessment; disarrangement of the honeycomb due to epidermal flattening secondary to epithelial and stromal proliferation caused by the infiltration of the pea shaped cells (red arrow heads). (B‒D) Dermo-epidermal junction and papilar dermis assessment; proliferation of “pea-shaped” atypical epithelial cells (red arrowheads) forming lobulated (light-blue arrows) and cerebriform nests (light green arrows).

In this report, we describe and correlate clinical, dermatoscopic, ivRCM, and histopathologic features of a cutaneous mixed tumor located on the nose. Since this is an infrequent tumor, there are no large series published; however, the literature presents an extensive description of clinical and histopathological characteristics. Clinically, CMT, or chondroid syringoma, is an adnexal benign painless tumor of nodular morphology, skin-colored to erythematous, with slow vertical growth, and frequently located in the head and neck.^4^ Based on these characteristics, the main clinical differential diagnoses include epidermal or subdermal cysts, trichoepithelioma, dermatofibroma, classic syringoma, and other adnexal neoplasms.^4^

Histopathological evaluation is essential for diagnosis due to the low specificity of clinical and dermoscopic findings (Fig. 4A–C). CMT is characterized by an epithelial component consisting of nests and cords of cuboidal cells with eosinophilic cytoplasm, and a mesenchymal component that may present myxoid, chondroid, fibrous, or adipose characteristics. Tubuloalveolar structures and ductal and/or keratinous cysts that exhibit apocrine or eccrine glandular differentiation are frequently observed.^1^, ^5^ The presence of cartilaginous tissue in the stroma is rare.^6^ The main histopathological differential diagnoses include apocrine adenoma, pilomatricoma, sebaceous cyst, and pleomorphic adenoma of the salivary glands.^4^

**Fig. 4:**
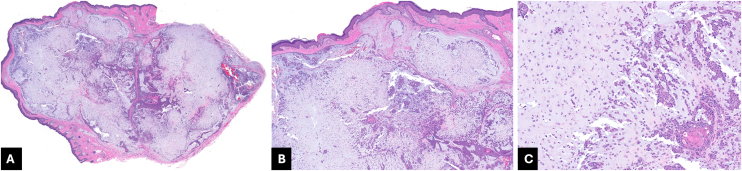
Histological (Hematoxylin & eosin) images of chondroid syringoma. (A) Hematoxylin & eosin, 20×; (B) Hematoxylin & eosin, 50×; (C) Hematoxylin & eosin, 200 × . The tumor displays a proliferation of epithelial cells within a rich chondroid and myxoid stroma. The cells are organized in cords and nests, and exhibit ovoid nuclei, small nucleoli, and pale cytoplasm. Focal keratinization is also present.

Dermoscopically, there are no pathognomonic signs for CMT, largely due to the scarcity of reported cases. However, some of the characteristics described in our case overlap with those of other adnexal tumors. The systematic review by Di Guardo et al. of 241 articles described recurrent dermatoscopic features identified in Chondroid syringoma, including whitish areas without structure, telangiectasias, milium-type cysts, and erythematous and/or homogeneous bluish areas.^7^ Additionally, unique presentations such as marbled appearance, pigmented macules, and vessels in signet ring configuration were also described. These rare presentations make the diagnosis challenging. Therefore, the main dermoscopic differential diagnoses include basal cell carcinoma, nodular hidradenoma, trichoepithelioma, pilomatricoma, and sebaceoma.^7^

Diagnosing CMT can be challenging because clinical and dermoscopic findings are nonspecific. The use of ivRCM has been scarcely reported, likely due to the technique's limitations related to cell visualization depth. Although the diagnosis of these tumors depends fundamentally on histopathological analysis, ivRCM is a rapid and non-invasive tool that can provide architectural and cellular features. In this case, ivRCM showed an atypical honeycomb pattern with a proliferation of “pea-shaped” atypical epithelial cells forming cord-like nests, supporting the decision to excise a progressively growing tumor.^1^, ^3^ We conclude that clinical features, together with dermoscopy and RCM, can be used as complementary examinations to guide a presumptive diagnosis and support the decision to excise. Nevertheless, more studies are required to better characterize this entity, ultimately avoiding unnecessary excisions.

## ORCID ID

Nelson Lobos-Guede: 0000-0003-0818-023X

Sofía Jiménez-Castillo: 0009-0005-8458-2363

Ingrid Plass: 0009-0001-3774-0390

Magdalena Delgado: 0000-0002-9384-8427

Alex Castro: 0000-0003-4431-5293

## Financial support

None declared.

## Authors' contributions

Nelson Lobos-Guede: Approval of the final version of the manuscript; critical literature review; manuscript critical review; preparation and writing of the manuscript.

Fernanda Ramírez-Bravo: Critical literature review; manuscript critical review; preparation and writing of the manuscript.

Sofía Jiménez-Castillo: Critical literature review; manuscript critical review; preparation and writing of the manuscript.

Ingrid Plass: Critical literature review; manuscript critical review; preparation and writing of the manuscript.

Magdalena Delgado: Critical literature review; manuscript critical review; preparation and writing of the manuscript.

Alex Castro: Critical literature review; manuscript critical review; preparation and writing of the manuscript.

## Research data availability

Does not apply.

## Conflicts of interest

None declared.
